# Quantifying Regional Vegetation Cover Variability in North China during the Holocene: Implications for Climate Feedback

**DOI:** 10.1371/journal.pone.0071681

**Published:** 2013-08-20

**Authors:** Guo Liu, Yi Yin, Hongyan Liu, Qian Hao

**Affiliations:** College of Urban and Environmental Sciences, Peking University, Beijing, China; University College London, United Kingdom

## Abstract

Validating model simulations of vegetation-climate feedback needs information not only on changes in past vegetation types as reconstructed by palynologists, but also on other proxies such as vegetation cover. We present here a quantitative regional vegetation cover reconstruction for North China during the Holocene. The reconstruction was based on 15 high-quality lake sediment profiles selected from 55 published sites in North China, along with their modern remote sensing vegetation index. We used the surface soil pollen percentage to build three pollen-vegetation cover transfer models, and used lake surface sediment pollen data to validate their accuracy. Our results showed that vegetation cover in North China increased slightly before its maximum at 6.5 cal ka BP and has since declined significantly. The vegetation decline since 6.5 cal ka BP has likely induced a regional albedo change and aerosol increase. Further comparison with paleoclimate and paleovegetation dynamics in South China reproduced the regional cooling effect of vegetation cover decline in North China modelled in previous work. Our discussion demonstrates that, instead of reconstructing vegetation type from a single site, reconstructing quantitative regional vegetation cover could offer a broader understanding of regional vegetation-climate feedback.

## Introduction

The need for reducing uncertainty in global climate change predictions has highlighted the importance of integrating model evaluations, on-site and remote sensing monitoring, paleoecological investigation, and small–scale manipulative experiments [1], [2]. Paleoecological data, with their unique advantage in addressing earth system processes at large temporal scales and under extreme conditions, could offer unique insights in this respect [2], [3]. The application of paleoecological data in reconstructions, however, requires the conversion from geological proxies to time-series of reconstructed variables [4].

Vegetation type can be reliably interpreted from pollen records through palynological methods [5], [6], [7], but is hard to quantify and therefore difficult to compare with model simulations or remote sensing monitoring. Modern remote sensing techniques, on the other hand, have allowed the evaluation of vegetation through numerical characteristics that offer important information for model simulations [8], [9]. Using similar palynological methods, with remote sensing data as a modern analogue, vegetation in the past could be reconstructed in a different manner, avoiding a discrete and discontinuous vegetation type reconstruction while providing a detailed description of past vegetation that is comparable with model output. Although it remains uncommon, this approach to vegetation reconstruction has been successfully exploited in some former studies [10], [11], [12].

While paleoenvironmental reconstruction could provide evidence of ecological and climatological processes at large temporal scales, distinguishing variations due to regional trends from those due to local heterogeneity is difficult and thus interpretations are often restricted to being local-scale and case-specific when based on one single site [4], [13]. By combining data from multiple sites, vegetation reconstruction at a regional scale could provide a broader insight into regional scale earth system process: this has already been achieved in many data-rich regions such as Europe [6] and North America [14]. Published late-Quaternary pollen records from northern Asia have increased rapidly in number over the past several decades [15], [16], [17]; in North China, however, reliable pollen data sites with relatively high resolution remained scarce [10] until the collection of higher quality data in recent years (Tab. S1). In this study, we selected 15 sediment profiles with relatively high resolution and reliability from 55 published sites in North China (Table S1), and reconstructed the regional vegetation cover changes during the Holocene using a remote sensing vegetation index (NDVI, normalized deference vegetation index) as a modern analogue. This quantitative regional reconstruction provided an opportunity to examine millennial-scale regional vegetation-climate interaction, and supported the hypothesis of a cooling effect of vegetation decline [18], [19] [20] through comparison with previous model simulations [1].

## Data and Methods

### 1. Pollen dataset

One major obstacle to past vegetation reconstruction is the deficiency of modern lake sediment analogues. Lake sediment profiles are widely used due to their ability to preserve signals of past environmental change; without these data, reconstructions are restricted to surface soil as the modern analogue for lake sediment profiles [21], [22], [23], with much uncertainty remaining with respect to the relationship between surface soil pollen and lake sediment pollen [24], [25]. In this study, both surface soil pollen data and lake surface sediment pollen data were utilized to render the reconstruction as reliable as possible: three simple pollen-NDVI transfer models were built from the modern surface soil pollen and modern NDVI, and were validated by the dataset from modern lake surface sediments. Finally, the most accurate transfer model was applied to the sediment profiles.

**Figure 1:**
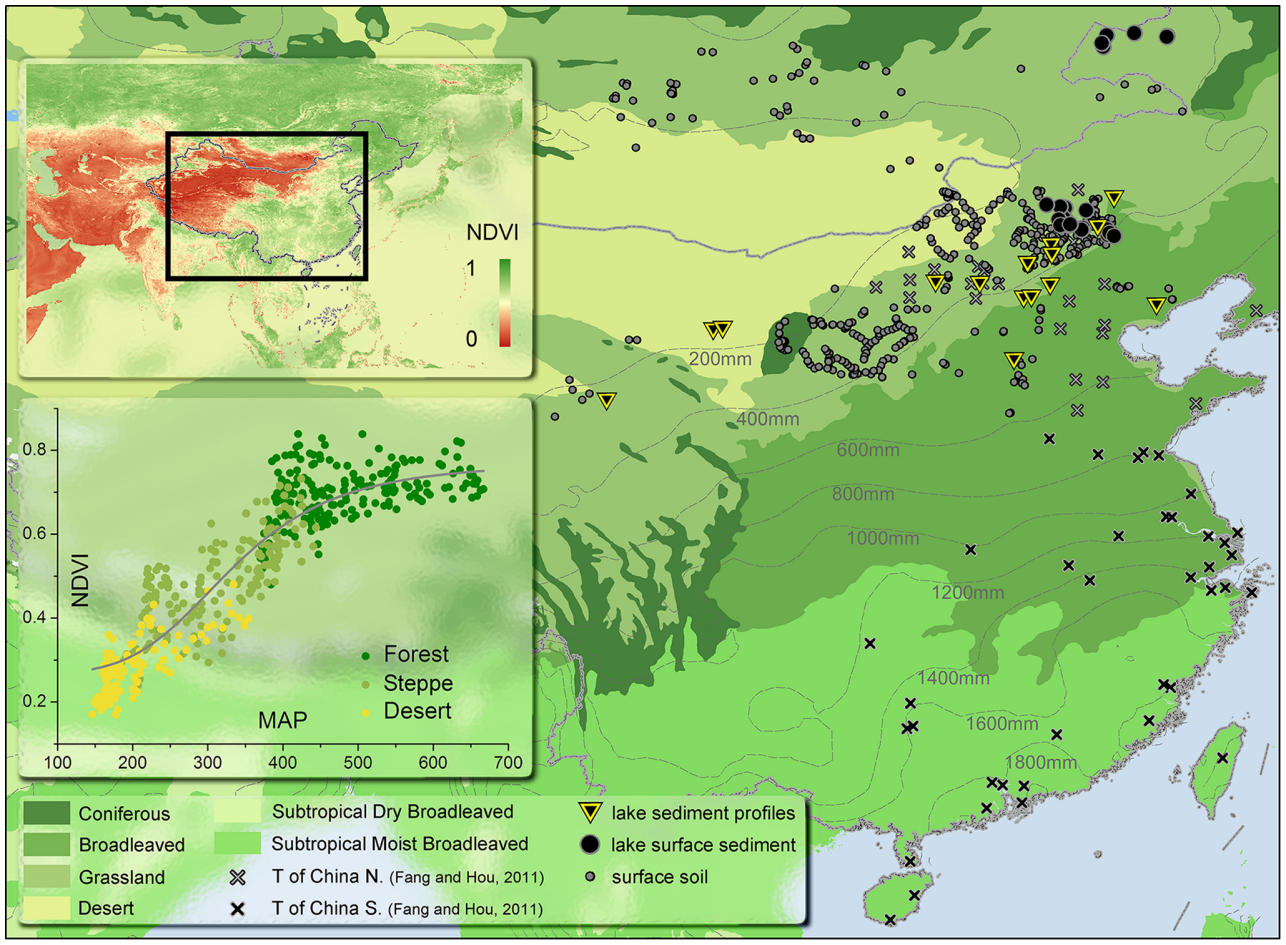
Sample locations and modern NDVI distribution. Modern NDVI data were acquired by averaging data for August from 1982–2006 of the GIMMS dataset. NDVI in South China is homogeneous at a high level, while that of North China varies widely with precipitation. Samples of NDVI-MAP relationships were randomly chosen from grid points with natural vegetation in our study area (North China) and fitted by a logistic curve. Sites with surface soil pollen, lake surface pollen and sediment profiles are distributed around the 400 mm isohyet; some sites with surface soil pollen samples are located in Mongolia, but in the same biome and precipitation regime. *T of China N.* and *T of China S.* indicate the paleotemperature records used in the temperature reconstruction of North China and South China [70], respectively.

A total of 461 published surface-soil pollen records from across North China were collated in this study, collected in regions with mean annual precipitation ranging from 100 to 700 mm (Fig. 1). Over 60 pollen taxa were found in these records, of which *Pinus, Betula, Quercus, Artemisia* and Chenopodiaceae appeared most frequently; these taxa have already been identified as indicators of vegetation type in our study area [26], [27]. Redundancy analysis (RDA) with all the major taxa in the soil pollen (Fig. 2a in [35]) showed that these 5 taxa (especially Pinus, Betula and Quercus) could explain most of the geographical variation of the soil pollen dataset; in addition, comparing the R^2^ of models constructed by these 5 most common taxa or by 18 common taxa showed that the difference (<0.05) is non-significant. While making use of more information from the pollen data, using more pollen types could at the same time increase the risk of model over-fitting, especially by artificial neural networks. In addition, models will be sensitive to taxa that only occur in few samples, which could introduce bias and uncertainty in the reconstruction. Therefore, the percentages of these five taxa were chosen as dependent variables to establish pollen-NDVI models. Lake surface sediments (upper 5 cm) of 27 perennial lakes with low human disturbance were collected and analysed, to validate the surface-soil pollen-NDVI models constructed by surface soil samples. The model with the highest accuracy was then applied to the selected sediment profiles.

**Figure 2:**
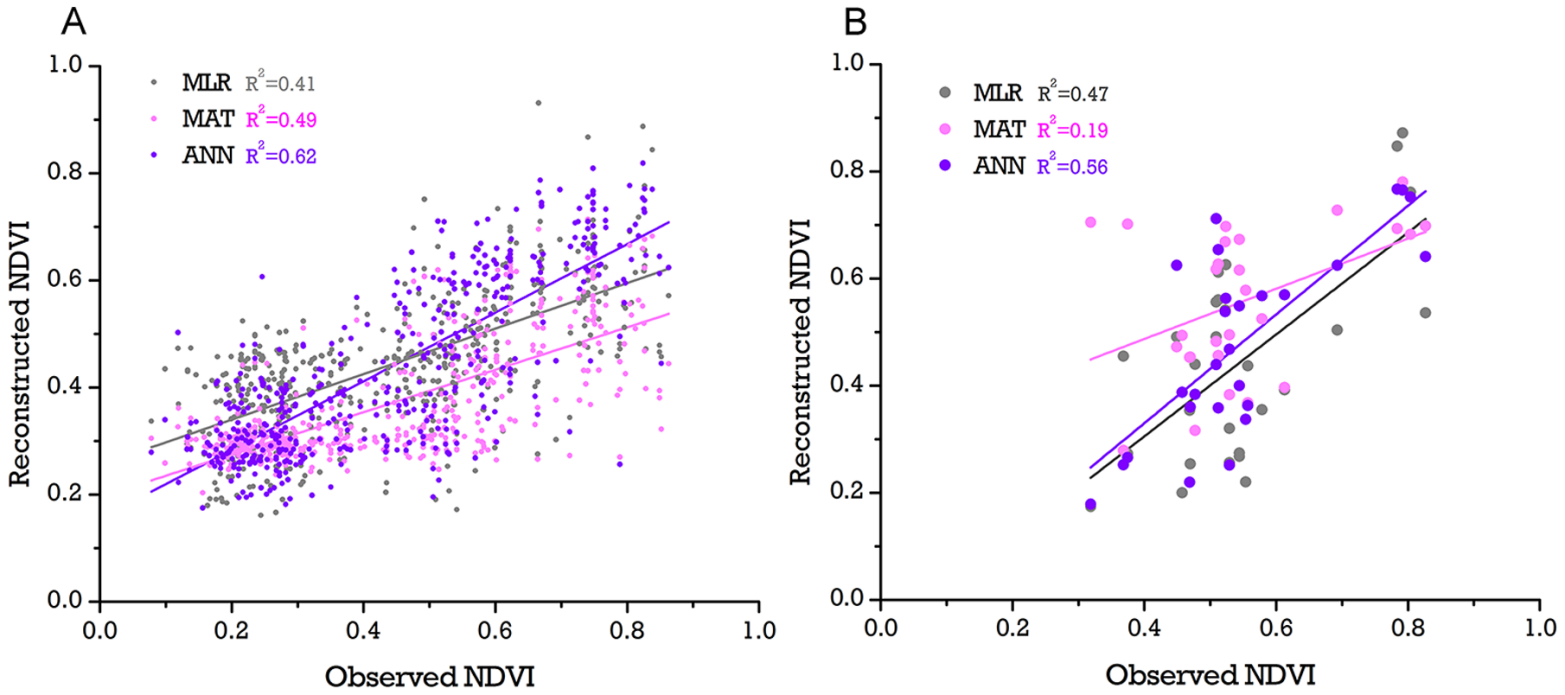
Result of model reconstruction and verification. In subplot A, LR, MAT and ANN all performed well in the model construction phase and passed the 0.01 significance level test; in subplot B, when verified by lake surface sediment pollen data, MAT failed to produce a reliable result. ANN was chosen for the reconstruction. All R^2^ data are the adjusted R square.

To select the most appropriate data from the 55 available profiles in or near our study area, three criteria were used: 1, the profiles cannot be too far away from the 27 lakes with surface sediment pollen analysis data, to guarantee the effectiveness of the validation; 2, the profiles must cover the last 10 ka, to guarantee the same number of samples for every period; and 3, the profile must have a high temporal resolution, as the trend in vegetation cover largely depends on the time period selected, thus a higher temporal resolution will decrease the uncertainty in period selection. Among all the 55 profiles, 34 were close to the 27 lakes used in this study; among these 34 profiles, 29 had an average resolution higher than 500a; and among these 29 profiles, 15 covered the last 10 ka. Proportions of the five chosen taxa were digitised from these 15 profiles and the corresponding vegetation cover was then reconstructed (Fig. 1). We then resampled the 15 reconstructed vegetation cover time series to a resolution of 200a because different profiles cover different time periods, such that resampling to a finer resolution allowed better use of the data. Finally, the 15 profiles were averaged to yield the regional vegetation cover changes.

### 2. Modern NDVI distribution patterns

The GIMMS NDVI (normalized difference vegetation index) dataset [28], [29], [30], running from 1982 to 2006 with a spatial resolution of 8 km×8 km, was used to calculate the NDVI of each sampling site. The lakes in our sediment pollen dataset had an average diameter of less than 1 km and the local impact of lakes on albedo and vegetation was thus ignored at the scale of an NDVI pixel. We then calculated the average of the yearly maximum NDVI values from 1982 to 2006 for each site with both surface soil and lake surface sediment samples in our analysis. Distributions of averaged NDVI and mean annual precipitation, as well as vegetation types, are plotted in Fig. 1.

### 3. Pollen-NDVI transfer models

Three pollen-NDVI models were constructed using pollen records in surface soil and were verified by corresponding records in the lake surface sediment, with the most accurate model being used to reconstruct NDVI from the pollen spectrum in the sediment cores. Models of pollen-NDVI relationships were built using an artificial neural network (ANN), the modern analogue technique (MAT) and linear regression (LR). Then the lake surface sediment pollen and associated NDVI dataset were applied to these models to verify their accuracy.

Firstly, we constructed a model of the relationship between the pollen spectra and NDVI by applying an ANN, which is a very flexible nonlinear method that is able to precisely simulate complex mapping [31]. In our study, the ANN used the percentages of *Pinus, Betula, Quercus, Artemisia* and Chenopodiaceae of each sample as input variables and the corresponding NDVI as the target variable. After experimenting with different parameter matching configurations, we chose three hidden layers with node numbers of 8, 10, 10 and the activation functions *tansig, logsig, logsig* (in Matlab R2009b), respectively.

Next we applied MAT, which has been widely used in vegetation reconstruction with pollen data [32], using the squared chord distance (SCD) as the index of similarity [33], [34]:
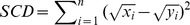
where SCD is the squared chord distance between two multivariate samples *X* and *Y*, and *x, y* are the proportions of species *i* in samples *X* and *Y*. SCD values can range from 0 to *n* (number of species), with 0 indicating pollen spectra identical to those of the samples being compared. For each fossil record, five samples with the smallest SCD (with the sample itself excluded) were selected from the surface soil pollen dataset and the corresponding NDVI was reconstructed by the weighted-average:



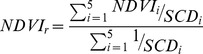
where 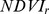 is the reconstructed NDVI, and 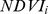 and 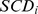 are the NDVI and squared chord distance of each sample.

The third method of reconstruction applied here was linear regression (LR). It can be assumed that for each taxon existing in a biome, every individual occupies a certain ratio of the area and contributes a corresponding value to the total NDVI; therefore, the relationship between total NDVI and the proportion of each taxon may have a linear component. LR has the significant advantage of being intuitive, with the respective parameters clearly showing the contribution of each taxon.

These three models of pollen spectra and NDVI, built from our surface-pollen dataset, could have been evaluated by means of the adjusted correlation coefficient and residual error between the estimated NDVI and observed NDVI. A proper approach, however, has to extend its accuracy into the fossil sediment pollen record. Therefore, we used our sediment pollen dataset to test the reliability of extending the models into the sediment pollen record by calculating the correlation coefficient between the simulated NDVI and observed NDVI of lake surface sediment samples (Fig. 2b).

All the above calculations were performed with the Matlab R2009b software.

### 4. Statistical analysis of the reconstructed NDVI

To maximise accuracy of the results, the 15 sediment profiles were selected to occupy the same time range (11.5 cal. ka BP to present) and to have comparatively high resolution (200 yr or higher). Each profile was resampled to 200 years temporal resolution before reconstruction. The reconstruction thus produced 15 comparable time series ranging from 11.5 cal ka BP to present. The stalagmite δ^18^O from a former study was sampled from Dongge cave in the Pacific monsoon region of China as a proxy for precipitation in the monsoon region, and was linearly correlated with the reconstructed vegetation cover. Meanwhile, the coarse sand (>63 μm) percentage from Anguli Nuur Lake in North China, located in the central-eastern region of our study area in the forest-steppe ecotone about 250 km northwest of Beijing, was used as an indicator of local soil coarsening, and was correlated with the reconstructed vegetation cover.

## Results

The ANN yielded the highest accuracy in the pollen-NDVI transfer model and was therefore used in our vegetation cover reconstruction. Each of the three models (ANN, MAT and LR) performed well in establishing the relationship between surface soil pollen and NDVI, with adjusted R^2^ values of 0.62, 0.49 and 0.41 (P<0.01), respectively. In the validation by lake surface sediment pollen, only ANN and LR continued to produce reliable results, with adjusted R^2^ values of 0.56 and 0.47 (p<0.01), respectively. Therefore, only the reconstruction by ANN was used in the following analysis.

**Figure 3:**
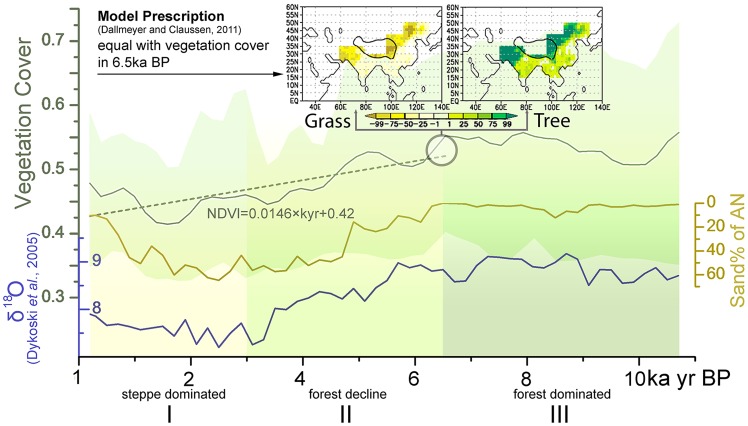
Mean reconstructed vegetation cover of each profile for 1–2, 6–7 and 9–10 ka BP. The circle diameters show the mean values of the reconstruction within 1 ka, while modern NDVI and modern MAP are plotted in the background. Because the heterogeneity between sites is much larger than the variation, the diameters of the circles were plotted according to the relative values compared to the mean value of the entire time series at each site.

**Figure 4:**
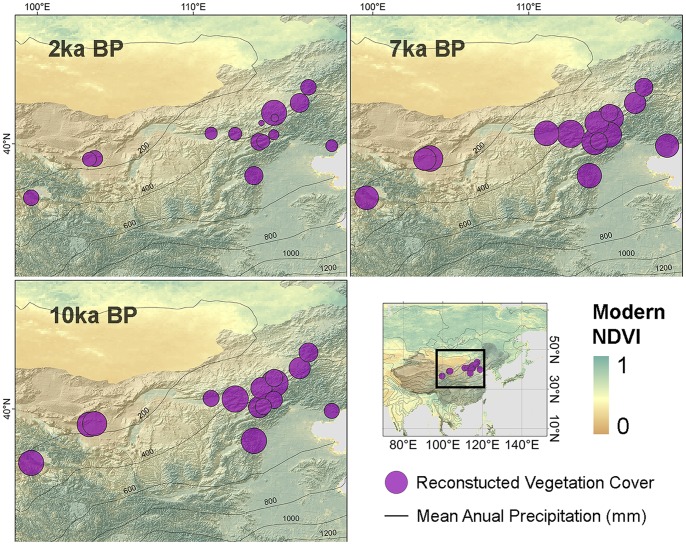
Reconstructed vegetation cover, related factors and prescribed values in previous studies. *Vegetation Cover* shows the reconstructed NDVI averaged from 15 sequences, with linear regression after 6.5 ka yr BP showing a decrease of 0.13. The light green area indicates the upper quartile and lower quartile of the 15 sequences, indicating that areas with higher NDVI experience larger fluctuations and were the major contributors to the vegetation decline in *Period II*. Sand% of AN shows the coarse sand (>63 μm) percentage in sediment cores from Anguli Nuur (inversely scaled). Stalagmite δ^18^O of Dongge Cave (as δ^18^O, values are negative), coarse sand percentage of Anguli Nuur and reconstructed NDVI are significantly correlated with each other. Prescribed values in the model simulation by Dallmeyer and Claussen (2011) coincide with the vegetation cover at 6.5 cal ka BP as reconstructed here; the colour bar shows the percentage of grass/trees in comparison to the modern vegetation distribution.

Reconstruction results show that vegetation cover in North China increased slightly before its maximum at 6.5 cal. ka BP and has since declined significantly (Fig. 3). The Holocene can be divided into 3 periods according to the averaged results from 15 profiles with 200-year resolution, as follows. *Period III* (11.5–6.5 cal. ka BP): most profiles in this period were identified as forest in former studies; vegetation cover fluctuated greatly but showed an overall slight increase before reaching its maximum at 6.5 cal. ka BP. *Period II* (6.5–3 cal. ka BP): a greater number of profiles were identified as steppe or forest steppe in this period, regional vegetation cover declined significantly from 0.57 to approximately 0.46. *Period I* (3 cal. ka BP- present): most profiles were identified as steppe, but the decline in Period II ended and there was even a slight increase in the most recent 1 cal. ka BP. Although 15 profiles are not sufficient to map the distribution of vegetation cover, we calculated and plotted the mean reconstructed vegetation cover of each profile for 10–9, 7–6 and 2–1 cal. ka BP (Fig. 4) to indicate the state of each period. Upper and lower quartiles in each 200-yr period were also calculated, showing the difference within sub-regions with different levels of vegetation cover.

## Discussion

### 1. Vegetation cover changes and their driving forces

The number of profiles adequate for regional vegetation reconstruction is directly related to the indicating area of lake sediment pollen and the vegetation heterogeneity in the studied area. Former studies conducted in small lakes with an assumption of neutral atmospheric conditions in regions dominated by forest generally showed that the source area of sediment pollen is within a radius of 1 km [35], [36], [37], [38]. With a source area of this scale, 15 profiles would be a poor representation of this region; however, in areas with a relatively open landscape, e.g. North China and the North American Central Plain, strong winds prevail through most of the year causing long distance transportation of dusts [39], which carry 20–1000 μm particles including pollen grains [40]. Combining surface soil pollen data and remote sensing data in a similar fashion to this study, research in eastern North America has yielded indicating areas with half-widths of 25–75 km [41]. Using a similar method, we have also calculated the indicating area of surface soil pollen and lake surface sediment pollen (corresponding to the lake sediment profiles used in reconstruction) in our study area (Yin et al., unpublished manuscript) and identified an average indicating area much larger than that of traditional estimates (half-widths of 20 km for surface soil, and over 100 km for surface lake sediment.

Although we could not directly quantify the heterogeneity of paleo-vegetation cover in our study area from the 15 profiles, we were able to assess whether the variability in these profiles could cause uncertainties in our results, and we indirectly evaluated the uncertainty related to vegetation cover heterogeneity. As our conclusions were based on the trend in vegetation cover, the relative change in vegetation cover was more important than the actual value of reconstructed vegetation cover in our study. Despite the different magnitudes of variability, the upper quartile, the lower quartile and the average of the 15 sequences showed the same trends (Fig. 3), indicating that the difference between profiles has little influence on our conclusion. Thus we believe that the 15 profiles are representative of the geographical range they cover. However, as the 15 profiles did not include sediment from the Loess Plateau, which has markedly different vegetation, the reconstructed vegetation cover only includes the regions of North China outside of the plateau.

Average results from the 15 profiles showed that vegetation cover in North China increased slightly from 11 cal. ka BP and reached its maxim at 6.5 cal. ka BP, before decreasing significantly. Calculation of the upper and lower quartiles in each 200-year period revealed the range in levels of vegetation cover between different regions: accordingly, the overall trends in vegetation cover were mainly attributed to changes in regions with high vegetation cover (mostly forest), while regions with low vegetation cover (mostly grassland and desert) remained relatively stable. Our results coincide well with those of former studies of Holocene vegetation types in North China (Tab. S1): in *Period III*, when vegetation cover fluctuated greatly with a slight overall increase, the vegetation type was mainly forest; in *Period II*, vegetation cover declined significantly and changed from forest-dominated to grassland-dominated at most sites; in *Period I*, the decline of vegetation cover slowed and North China was dominated by grassland.

Regional reconstruction of vegetation cover provides an opportunity for studying regional driving forces of vegetation cover, and the partial correlation between reconstructed temperature and stalagmite δ^18^O has revealed precipitation as the main driving factor of vegetation cover change. Precipitation in the Pacific monsoon regions of China during the Holocene could be indicated by the stalagmite δ^18^O proxy from Dongge Cave [42], [43]. Linear regression shows that this proxy is significantly and negatively correlated with the reconstructed vegetation cover in North China (R^2^ = 0.701, P<0.01), indicating the influence of precipitation on vegetation (a lower δ^18^O indicates higher precipitation). However, a potential risk of comparing the stalagmite δ^18^O from Dongge Cave with results from our reconstruction is the uncertainty in the δ^18^O interpretation. Although former studies have shown that δ^18^O of stalagmites in the region of Dongge Cave can be interpreted reliably as an indicator of the overall precipitation at a continental scale [43], [44], it has been argued that δ^18^O may also be influenced by temperature [45], [46]. Studies based on modern observations in North China, however, have shown that precipitation dominates vegetation growth while temperature only plays a minor role [47], [48], [49]. Thermal regulation of vegetation has been found in areas with vegetation decline due to warming-induced drought [48], [49], [50], which casts further doubt on the relationship between decreasing temperature and declining vegetation cover through the Holocene [51]. In addition, we conducted a model simulation using the ORCHIDEE dynamic global vegetation model (DGVM) to test the influence of precipitation and temperature on vegetation cover in this region, and have also found that precipitation strongly controls vegetation cover in North China, while temperature has a relatively weak influence (Liu *et al*., unpublished data).

### 2. Possible impacts of vegetation cover change

While vegetation cover is mainly controlled by precipitation, significant change on a regional scale could in return have impacts on regional climate. Among these impacts are the decrease in albedo and the increase of aerosol production. The degradation from forest to grassland or from grassland to desert is accompanied by an increase in albedo [52], [53]; in addition, NDVI, indicating vegetation cover change in our study, has a linear negative relationship with albedo [54], [55], and its reconstruction implies the albedo increase in *Period I*. A more significant mechanism contributing to albedo changes is related to the transformation between vegetation types. In the winter of higher latitudes, grassland is entirely covered by snow whereas forest can penetrate through snow cover and generally remains exposed. Therefore, the transformation from forest to grassland could significantly change the albedo of the land cover [56], [57]. This transformation has taken place in the past 6500 years in regions with high vegetation cover, corresponding to those regions which have been mainly attributed to the overall vegetation cover change owing to their position in the upper quartile of the reconstruction.

The other possible impact of vegetation cover decline is the increase in aerosol production: soil dust plays an important role in the production of aerosol, and could significantly alter the regional radiation balance [58], [59]. Soil coarsening or sandification, the main source of soil dust, could be indirectly inferred from the coarse sand percentage in sediment records. Vegetation cover decline may lead to soil sandification and an increase in wind velocity, both of which can contribute to a higher coarse sand percentage in lake sediments. Changes in wind velocity are attributed to the shift from forest to grassland, with a corresponding reduction in surface roughness [60], [61]. The vegetation type derived from this core has, however, shown the dominance of grassland since 5.0 cal. ka BP [62], while the coarse sand percentage has increased in tandem with the regional vegetation cover decline since then (except for a decrease in the past thousand years that coincides with the recovery of vegetation cover) (Fig. 4). A connection between coarse sand percentage and monsoon intensity has been reported throughout the last glacial maximum, but has mostly been observed at the larger temporal scale of the glacial-interglacial cycle and on plateaus such the Chinese Loess Plateau [63], [64]. The fluctuation of monsoon intensity during the Holocene was, however, much milder; in addition, the location of Anguli Nuur lake within a basin reduces its sensitivity to monsoon-controlled changes in wind velocity. A more likely explanation is that the soil coarsening or sandification accompanied vegetation decline [65], [66], which can still be observed around Anguli Nuur Lake. Through the increase in dust transferred to the atmosphere, desertification could become a further source of aerosol [58], [59], [67]. The regional average vegetation cover is used in this study instead of that solely reconstructed from the sediment core of Anguli Nuur Lake due to the lower credibility of reconstruction from a single site. Although this coarse sand percentage is mainly a local indicator, the correlation reflects the relationship between regional vegetation cover decline and local soil sandification and implies the consequential increase of dust and aerosol.

**Figure 5:**
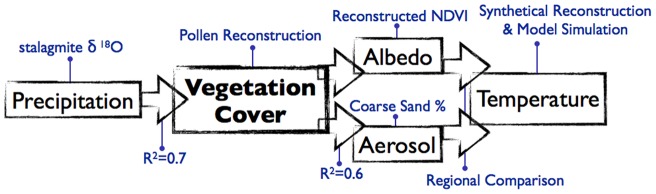
Mechanisms discussed in this study. Black boxes show the mechanisms discussed in the text, and are further explained by the blue annotations or the corresponding R^2^ in the linear correlation. While vegetation cover in North China is mainly controlled by precipitation, its decline in the past 6500 years might have led to the changes in both land cover albedo and aerosol production, with a resulting regional cooling effect, as model simulations in previous studies and the regional comparison in this study have shown.

Although the carbon emission accompanying the vegetation cover decline could have an impact at larger scales, both of the impacts discussed above suggest a cooling effect on regional climate (Fig. 5). Interactions between the regional vegetation-climate processes make it difficult to evaluate the overall effect of these feedbacks; however, regional comparison of paleoenvironmental reconstructions could provide evidence for the overall response over a long period of time, disregarding the uncertainties in the mechanisms' details. While the vegetation cover has declined significantly in North China since 6.5 cal. ka BP, vegetation in South China has been relatively steady and constantly dominated by forest [68], and its NDVI has remained saturated even to modern times [48]. Comparison between the vegetation records and temperature records in these two regions could thus offer insight into the overall thermal feedback of forest. Moreover, the vegetation-climate interaction in these regions has been discussed through a model simulation in a previous study [1], whose result implied a cooling effect of vegetation cover decline in North China that can be compared to our reconstruction.

As prescribed in the model, approximately 70% forest was added and 60% steppe removed in our study area, while vegetation change was comparably small in South China [1]. This prescription change, according to the modern distribution of NDVI (Fig. 1), is equivalent to a change of 0.1–0.15 in the NDVI of North China. Our result showed that, within the past 10,000 years, the vegetation cover consistent with these prescribed conditions occurred at 6.5 cal. ka BP (0.13 higher that modern times in NDVI, Fig. 3). Modelling results have indicated that North China was 1∼1.5 K warmer in the cold/dry season while South China was 0.25∼0.5 K warmer in the cold/dry season [1]. These results can then be compared to reconstructed temperatures for this period. Reconstructed temperatures around 6.5 cal. ka BP from single sites have shown a more significant warming, specifically 1.7–2.6 K, 3.0 K and 3.0–4.0 K for North China, and 1.0 K, 1.7 K and 3.0–3.5 K for South China [69], with a similar north-south contrast. Recent synthetic reconstructions of temperature in these two regions agree better with the model simulation, in which the linear trend in the temperature time series shows that North China was 1.8 K warmer and that South China (Southeast China and Central East China in the original publication) was 0.4 K warmer at 6.5 cal ka BP [70].

Situated in the Pacific monsoon region, both North China and South China are controlled by the same monsoon system (referred to as the SE Asian Monsoon in previous studies) [71], [72], and have shared similar climate histories during the Holocene. The differences in their temperature changes could be attributed, at least partially, to the contrasting vegetation cover changes. An opposing view could be that the vegetation cover decline was a result of the temperate change. However, as discussed in *Section 4.1.*, warming generally intensifies the water deficit of vegetation in North China [73], [74]; therefore it is unlikely that the decrease in temperature could have led the decline in vegetation cover of North China. Although agreement between model simulation and the mechanisms we have suggested cannot rigorously prove the causal-consequence relationship between vegetation cover and temperature, it strongly supports former modelling studies and clearly increase support for the existence of this feedback. While global warming due to greenhouse gas emissions is commonly regarded as a serious issue for the future, this cooling effect might partially compensate the decrease in carbon sinks and thus counteract the effects of global warming. Estimates of the intensity of this feedback are evidently critical for the precision of model simulations.

### 3. Prospective regional vegetation cover reconstruction

The importance of integrating models and paleoecology data, in both the process of constraining inverse modelling and in the structure adjustment in forward modelling, has been recognized with the development of mechanistic, comprehensive and complex structures in ecological models and with the rapid growth of data in paleoecology [3]. As many have argued before, the assimilation of proxy data into models has become important for the improvement of both palaeovegetation reconstructions and model simulations [75], [3]. As a result, while ecological models have been developed in preparation for the data assimilation application, palaeoclimate and palaeovegetation are being reconstructed in a manner that can be directly utilized by models [76], [77], [78], and intensive integration of paleoecological data and model simulations has recently been achieved [79], [80]. Reliable methodologies and a paradigm for single-site based vegetation type reconstructions has been developed and recognized, but the implications of such reconstructions are, however, often site-specific and locally-restricted, with results that are difficult to quantify. We have shown in this study that regional vegetation cover reconstruction, with the help of newly developed vegetation indices in remote sensing, could offer new insights in earth system models.

As a trial of this approach, we discuss the cooling effect of vegetation cover decline, which has been crucial not only because it might counteract the organic carbon release, but because it could also result in a regional overall cooling [19], [20]. This is in contrast to the long-held view that afforestation, which opposes the vegetation cover decline, can alleviate global warming [81], [82]. Previous observations, however, were conducted during comparatively short periods of time [19], [43], [83], which might be insufficient for a significant change in vegetation cover to take place. The long temporal scales in paleoecological studies are therefore valuable for the evaluation of similar processes. An ideal regional vegetation cover reconstruction would have a spatial resolution that could demonstrate the pattern of vegetation cover distribution, and could provide boundary conditions or driving parameters for model simulations. In North China, this could be achieved with the rapid accumulation of paleoecological data; however, at present, in this study the regional spatial pattern of vegetation cover could not be obtained reliably based on 15 profiles (which were selected out of 55 profiles in this region according to the quality control criteria) even if calibration with the modern vegetation cover pattern is taken into account. As a result, regional averaging was a compromise necessary in the comparison between results from the model and those from the reconstruction. Nevertheless, in view of the data-rich enterprise that palaeoecology has become, we here present a method of vegetation reconstruction that yields regional vegetation cover instead of vegetation type, and which is more suitable for addressing regional-scale questions and model-data comparison. Our discussion has demonstrated that this switch in the reconstruction objective could help in validating results from models, as well as in understanding the mechanisms underlying earth system process on a larger scale.

## Conclusions

In this study we quantitatively reconstructed the Holocene vegetation cover changes in North China from multiple pollen records, using the modern NDVI as an index. The results shows that:

1. Vegetation cover in North China increased slightly after 11 cal. ka BP and reached its maximum at 6.5 cal ka BP, but has decreased significantly since then. This change was mainly attributed to vegetation dynamics in regions with a high vegetation cover, while regions with low vegetation cover changed relatively little.
2. Vegetation cover decline in North China was mainly controlled by precipitation changes in the Pacific Monsoon region; this vegetation decline could have then induced local soil coarsening and a consequential increase in the production of aerosol.
3. One important implication of this result is its support of a thermal feedback of vegetation cover change as reported in former modelling studies, suggesting that the vegetation cover changes in North China could have had an overall cooling effect on this region during the late Holocene, owing to their alteration of albedo and aerosol production.
4. Combining multiple profiles and using modern vegetation indices as a modern analogue could yield a quantitative reconstruction of regional vegetation cover, which could be used in validating results from models as well as understanding ecological process on a larger scale. This advantage will become even more notable with the rapid accumulation of paleoecological data.

## Supporting Information

Table S1 Site description of the 15 selected sediment profiles in North China. This table contains the references and basic information of the 15 profiles used for vegetation reconstruction in this study.
(DOC)
