# Supplementary material for: Quantifying Regional Vegetation Cover Variability in North China during the Holocene: Implications for Climate Feedback
Source: PLoS One. 2013 Aug 20;8(8):e71681. doi: 10.1371/journal.pone.0071681 (PMC3748107; doi:10.1371/journal.pone.0071681)
Supplement: Table S1 — Site description of the 15 selected sediment profiles in North China. This table contains the references and basic information of the 15 profiles used for vegetation reconstruction in this study. (DOC) [file pone.0071681.s001.doc]

**Table S1 Site description of the 15 selected sediment profiles in North China**

| **No.** | **Name** | **Latitude**  **(°N)** | **Longitude**  **(°E)** | **DEM**  **(m a.s.l.)** | **MAP**  **(mm)** | **MAT**  **(°C)** |
| --- | --- | --- | --- | --- | --- | --- |
| 1 | Sanjiaocheng | 39.00 | 103.33 | 1325 | 105.2 | 7.7 |
| 2 | Zhuyehu | 39.05 | 103.67 | 1312 | 105.3 | 7.7 |
| 3 | Qinghai | 36.53 | 99.60 | 3200 | 345.9 | 0.3 |
| 4 | Huangjiabao | 40.57 | 115.15 | 601 | 347.2 | 5.7 |
| 5 | Anguli Nuur | 41.33 | 114.36 | 1311 | 369.5 | 2.6 |
| 6 | Chasuqi | 40.67 | 111.13 | 1011 | 372.6 | 6.4 |
| 7 | Taipusi | 41.98 | 115.18 | 1491 | 382.4 | 1.6 |
| 8 | Bayanchagan | 41.65 | 115.21 | 1370 | 385.4 | 1.6 |
| 9 | Daihai | 40.63 | 112.68 | 1237 | 386.5 | 3.6 |
| 10 | Xibao | 40.12 | 114.22 | 920 | 391 | 6.6 |
| 11 | Yujiagou | 40.15 | 114.48 | 855 | 393 | 6.4 |
| 12 | Sanyihuangtu | 43.62 | 117.38 | 1473 | 396.9 | -1.7 |
| 13 | Xiaoniuchang | 42.62 | 116.82 | 1411 | 402.1 | -0.3 |
| 14 | Nianziguan | 37.95 | 113.88 | 794 | 504.2 | 11.8 |
| 15 | Yanshengang | 39.87 | 118.87 | 58 | 656.9 | 10.5 |

**References**

1. Chen F, Cheng B, Zhao Y, Zhu Y, Madsen D (2006) Holocene environmental change inferred from a high-resolution pollen record, Lake Zhuyeze, arid China. The Holocene 16, 675-684.

2. Li Y, Wang N, Morrill C, Cheng H, Long H, Zhao Q (2009) Environmental change implied by the relationship between pollen assemblages and grain-size in NW Chinese lake sediments since the Late Glacial. Rev. Palaeobot. Palynol. 154, 54-64.

3. Zhao Y, Yu Z, Chen F (2009) Spatial and temporal patterns of Holocene vegetation and climate changes in arid and semi-arid China. Quat. Int. 194, 6-18.

4. Sun L, Xu Q, Yang X, Liang W, Sun Z (2001) The vegetation and environment in Xuanhua Basin after deglacial. J. Geomech. 7, 303-308. (in Chinese with English abstract)

5. Yin Y, Liu H, He S, Zhao F, Zhu J, Wang H, Liu G, Wu X (2011) Patterns of local and regional grain size distribution and their application to Holocene climate reconstruction in semi-arid Inner Mongolia, China. Palaeogeog., Palaeoclim., Palaeoecol. 307, 168-176.

6. Wang F, Song C, Sun X (1999) Palynological record of paleovegetation change during the Holocene at North Tumd Plain in Inner Mongolia, China. Chin. Geog. Sci. 9, 87-91.

7. Huang F, Lisa K, Xiong F, Huang F (2005) Holocene grassland vegetation, climate and human impact in central eastern Inner Mongolia. Sci. China (Ser. D) 48, 1025-1039.

8. Jiang W, Guo Z, Sun X, Wu H, Chu G, Yuan B, Hatté C, Guiot J (2006) Reconstruction of climate and vegetation changes of Lake Bayanchagan (Inner Mongolia): Holocene variability of the East Asian monsoon. Quat. Res. 653, 411-420.

9. Xu Q, Xiao J, Toshio N, Yang X., Zhen Z, Liang W, Li Y, Yoshio I (2004) Vegetation Succession and Climate Change in Daihai Basin Since Holocene: Evidence in Palynology. J. Glaciol. Geocryol. 26, 73-80. (in Chinese with English abstract)

10. Wang Y, Wang S, Jiang F, Tong G (2003) Pollen Analysis of Yangyuanxipu Profile. J. Geomech. 9, 171-175. (in Chinese with English abstract)

11. Xia Z, Chen F, Chen G, Zheng G, Xie F, Mei H (2001) [Environmental background of evolution from the paleolithic to neolithic culture in Nihewan Basin, North China](http://www.springerlink.com/index/C785045514074039.pdf). Sci. China (Ser. D) 44, 779-788.

12. Wang Y, Wang S, Zhao Z, Qin Y, Ma Y, Sun J, Sun H, Tian M (2006) Vegetation and Environmental Changes in Hexigten Qi of Inner Mongolia in the Past 16000 Years. Acta Geosci. Sin. 26, 449-453. (in Chinese with English abstract)

13. Liu H, Cui H, Huang Y (2001) Detecting Holocene movements of the woodland–steppe ecotone in North China using discriminant analysis. J. Quat. Sci. 16, 237-244.

14. Yang X, Xu Q (1999) Vegetation succession in Taihang Mountains since Last Glacial. Geog. Territ. Res. 15: 81-88. (in Chinese with English abstract)

15. Kong Z, Xu Q, Yang X, Sun L, Liang W (2000) Vegetation change from alluvium pollen analysis in the Yinma River Basin of Hebei Province. Chin. J. Plant Ecol. 24, 724-730. (in Chinese with English abstract)
